# Introduction: Practical Social and Industrial Research (PSIR) Symposium 2015

**DOI:** 10.1186/2193-1801-4-S2-I1

**Published:** 2015-11-27

**Authors:** Ronald Chi-kit Chung

**Affiliations:** Vocational Training Council, Hong Kong

The Practical Social and Industrial Research (PSIR) Symposium was scheduled to take place in the Hong Kong Institute of Vocational Education (IVE), Morrison Hill, Hong Kong, on 27 November 2015.

The Symposium was organised with the intent of bringing together vocational education and training (VET) practitioners from different subject areas possessing diverse expertise, who were involved in carrying out practical social research or developing industrial applications based on existing technologies. The aim of this Symposium was to create an environment stimulating interdisciplinary interactions, to present latest PSIR achievements and demonstrate current knowledge.

The PSIR Symposium 2015 featured the theme of "Taking Skills to the Next level" and consisted of two keynote speeches, three parallel oral presentation sessions and a poster presentation session. We were honoured to have invited two renowned speakers to share their experience and insights of conducting PSIR activities. One keynote speaker, Prof. Georges Halpern from the City University of Hong Kong, shared with the delegates the excitement of conducting applied research and its practical implications. The other keynote speaker, Mr. Eric Yim from POSH Office Systems (HK) Limited, revealed his experience in driving business success through innovation. Besides the profuse scientific programme, delegates were also able to listen to the sharing of various VET practitioners in securing external grants and their experience in pursuing externally-funded PSIR activities. The Symposium also hosted an exhibition for showcasing VET students' outstanding works of applied research.

Practical social research refers to studies that offer new perspective to address topical social and cultural issues, whereas applied industrial research refers to the development and application of technology and know-how to produce new products, new processes, or new applications for industries. For both cases, the element of "practice" in either the methodology or the research outcome is emphasised. The PSIR Symposium has been organised since 2014 as a pioneer Symposium combining social, cultural and technological applied research. Last year, the Symposium attracted over 180 delegates coming from a variety of disciplines to share their experience in solving current social problems and industrial challenges in practice. Similar to the previous event, the PSIR Symposium 2015 provided delegates with an opportunity to expand their networks, meet new people and nurture new partnerships and collaborations.

Future plans: The PSIR Symposium is a regular annual event organised by the Vocational Training Council in Hong Kong. Potential focus areas in 2016 will include vocational education and training, social innovation for ageing population, and environment and sustainability.

